# Historical and Continued Colonial Impacts on Heart Health of Indigenous Peoples in Canada: What’s Reconciliation Got to Do With It?

**DOI:** 10.1016/j.cjco.2021.09.010

**Published:** 2021-09-24

**Authors:** Annette Schultz, Thang Nguyen, Moneca Sinclaire, Randy Fransoo, Elizabeth McGibbon

**Affiliations:** a College of Nursing, Rady Faculty of Health Sciences, Helen Glass Centre for Nursing, University of Manitoba (UM), Winnipeg, Manitoba, Canada; b St Boniface Research Centre, St. Boniface General Hospital, Winnipeg, Manitoba, Canada; c Cardiac Sciences Manitoba, Asper Clinical Research Institute, St. Boniface General Hospital, Winnipeg, Manitoba, Canada; d Max Rady College of Medicine, Rady Faculty of Health Sciences, University of Manitoba, Winnipeg, Manitoba, Canada; e Rankin School of Nursing Faculty of Health Sciences, Faculty of Health Sciences, St. Francis Xavier University, Antigonish, Nova Scotia, Canada

## Abstract

Colonization and enforced genocidal strategies have collectively fractured and changed Indigenous people by attempting to erase and dismiss their voices and knowledge. Nearly a decade ago, we were reminded by Dr Ku Young of the cardiovascular health disparities, in evidence among Indigenous people in Canada. compared with White people. He went on to say that beyond a biomedical understanding of this health status is the ongoing impact of long-standing marginalization and oppression faced by Indigenous people. Limited attention has been afforded to advance our understanding of these colonial impacts on Indigenous people and their heart health. This article contributes to our collective understanding of Indigenous people and their cardiac health by covering the following topics: layers of foundational truths of relevance to healthcare contexts and Indigenous people; a critical reflection of Western (biomedical) perspectives concerning cardiac health among Indigenous people; and materials from 2 studies, funded by the Canadian Institutes of Health Research, in which Indigenous voices and experiences were privileged concerning the heart and caring for the heart. In the final section, 3 topics are offered as starting points for self-reflection and acts of reconciliation within healthcare practice, decision-making, and research: reflections on self and one’s worldview; anti-racist healthcare practice; and 2-eyed seeing approaches to work within healthcare contexts. A common thread is the imperative for “un-silencing” Indigenous people’s voices, experiences, and knowledge, which is a requirement if addressing the identified cardiovascular health disparities is truly a health priority.

## Graphical abstract

**Figure:**
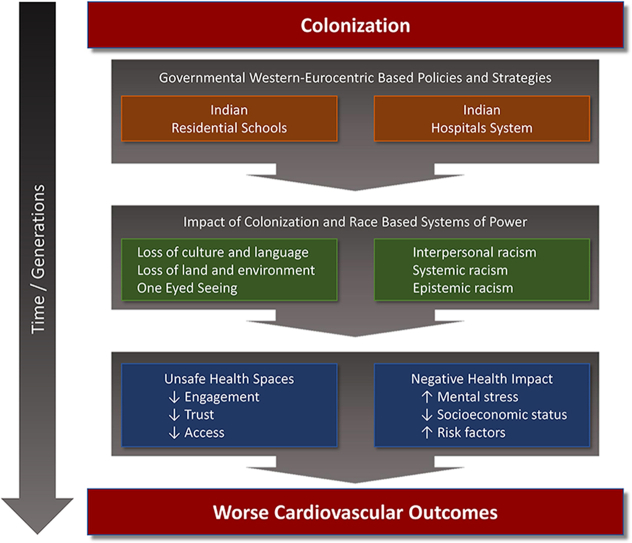

## Résumé

La colonisation et les stratégies génocidaires mises en œuvre ont concouru à diviser et à transformer les peuples autochtones dans une tentative d’effacement et de rejet de leurs voix et de leurs connaissances. Il y a près d’une décennie, le Dr Ku Young nous a rappelé les disparités en matière de santé cardiovasculaire, mises en évidence chez les peuples autochtones du Canada comparativement aux blanches. Il a ensuite déclaré que, outre la dimension biomédicale de cette situation sanitaire, l’effet de la marginalisation et de l’oppression de longue date des peuples autochtones est en cause et qu’il continue de se faire sentir. Peu d’attention a été accordée à l’avancement de notre compréhension des effets de la colonisation sur les peuples autochtones et leur santé cardiaque. Le présent article contribue à notre compréhension collective des peuples autochtones et de leur santé cardiaque en abordant : les strates de vérités fondamentales importantes dans le contexte des soins de santé et pour les peuples autochtones; les points de vue occidentaux (biomédicaux) sur la santé cardiaque des peuples autochtones, sous l’angle d’une réflexion critique; les données de deux études, financées par les Instituts de recherche en santé du Canada, où les voix et le vécu des autochtones ont été privilégiés en matière de santé cardiaque et au regard de ce qu’elle implique. Dans la dernière partie de notre article, les trois thèmes suivants sont présentés comme autant de points de départ d’une autoréflexion et de gestes de réconciliation en matière de prestation de soins de santé, de prise de décision et de recherche : réflexion sur soi-même et sa vision du monde; prestation de soins de santé antiraciste; approche à double perspective des soins de santé. Il est impératif de briser le silence et de « laisser s’exprimer » les voix, le vécu et les connaissances des peuples autochtones. Ce constat est un des fils conducteurs de notre analyse. Il faut agir en conséquence si tant est que l’élimination des disparités observées en matière de santé cardiovasculaire constitue vraiment une priorité sanitaire.

Colonization and enforced genocidal strategies changed our lives in profound ways and collectively fractured Indigenous people by attempting to erase and dismiss our knowledge. Even with this, we continue to understand that the heart is more than a physical organ but also it is its own living entity within the human body. It has its own way of having a relationship with the human body both inside, with all the other organs, and outside the body, with others, the land, and the creator. For us, the heart worked best with the mind, so much so that if the mind died, the heart could keep the body living; however, if the heart died, all else ceased to live. This is how powerful the heart is for us; we know if it is not looked after through relationships to other humans and the earth that it could be broken, die, or today, it can be replaced. We also know acknowledgement of the previous relationship must occur. Without that acknowledgement, “rejection” could happen. We also understand the different ways you see the heart, and we invite you to hear about and see how we view the heart. Our vision is that, together, new perspectives and knowledge can create a different path towards heart health.

*—Dr Moneca Sinclaire Nēhinan, (Cree) Health Researcher*

Nearly a decade has passed since Dr Ku Young published an article addressing cardiovascular health among Canadian Aboriginal people.^1^ He discusses the burden of cardiovascular disease among Aboriginal populations through biomedical perspectives and acknowledges that historical and ongoing oppressive marginalization of Aboriginal peoples underlies the growing health burden. Since then, Canadian health researchers’ contributions include reporting on rates of cardiovascular diseases, related treatments, cardiac risk factors, and health outcomes.^2^, ^3^, ^4^, ^5^, ^6^, ^7^, ^8^, ^9^, ^10^, ^11^, ^12^ Although contributions rooted in biomedical perspectives are expected, there is notably limited attention given to understanding the impacts of historical and ongoing colonization and racism.^13^, ^14^, ^15^ The growing burden of cardiovascular disease among Indigenous peoples in Canada has yet to be effectively addressed by drawing on evidence and perspectives derived from biomedical perspectives. Until systemic racism embedded in healthcare systems, including epistemic racism (see Box 1),^16^ are redressed, strategies that rely solely on biomedical perspectives likely will continue to be ineffective. We suggest that listening to Indigenous people’s voices, and working with their knowledge as valid evidence to inform healthcare decisions, could lead to novel strategies to address cardiovascular health among Indigenous people.

Box 1 **Defining the many faces of racism**

Racism is experienced as differential treatment, such as prejudice or discrimination, based on being a member of a racial group, which is commonly experienced by members of a minority racial group. Although multiple forms of racism have been identified, below we discuss 4 types, with the intent of illustrating diverse forms. The choices draw on Allan and Smylie’s report First People, Second Class Treatment^16^ (p.5).

**Interpersonal Racism:** Acts that occur between individuals; this is the most commonly talked about form of racism. Examples of these acts include discriminatory treatment in employment, healthcare delivery, and educational settings; they also include prejudice or demeaning acts that occur within any day-to-day interactions among 2 or more people. The severity of these acts can vary from ignoring or shunning, to poor treatment, to more overt and harsh acts such as name calling and physical or sexual violence. Raced-based “microaggressions” are also examples of interpersonal racism, which is defined as “brief or commonplace daily verbal, behavior and environmental indignities, whether intentional or unintentional, that communicates hostile derogatory, or negative racial slights and insults to the target person or groups.”^17^

**Systemic Racism**: race-based differential enactments through social systems, structures, and institutions, which can include stated criteria or conditions for an individual to be eligible or not. Although experienced by individuals within a group, evidence of systemic racism is found when reviewing practices, policies, and processes that reveal underlying, avoidable, and unfair inequalities across ethnic/racial groups. Systemic racism can serve to produce racialized inequality, but also be re/enacted when those in power (ie, policymakers, funders) fail to redress such inequalities. Systemic racism is often experienced as social exclusion and isolation, which also limits or prevents political and economic participation, or access to and participation in other social systems such as education or health.

**Epistemic Racism:** racialized acts that occur within the realms of knowledge—that is, asking whose knowledge and way of knowing is held up as superior and valid, vs the silencing and judging of another groups’ knowledge and ways of knowing as inferior, not to be considered valuable, and dismissed. In the context of Indigenous health, an example of epistemic racism (Sinclaire at al.)^18^ is that imposition of Western knowledge systems (biomedical science) has intentionally and unintentionally produced evidence to suggest inferiority of Indigenous people and their science (ways of knowing). Ongoing marginalization of Indigenous ways of knowing and practices within healthcare is a clear example of epistemic racism.

**Internalized Racism:** when an individual or group internalizes and accepts negative, stereotypical beliefs, attitudes, and ideologies about their inferiority.

Adapted from Allan and Smylie.^16^

Over the past 7 years of collaborations on the Debwewin—The Truth of Our Hearts study, the co-authors of the current article were privileged to listen to and learn from an Indigenous health researcher, Dr Moneca Sinclaire. For example, at a meeting, she shared that any health study concerning Indigenous people today will likely say less about Indigenous people and more about what happens when you colonize a group of people for centuries. The process of colonization is when an external group imposes their sociopolitical, economic, cultural, educational, and healthcare systems upon another group, along with an underlying intention of eliminating their ways through assimilation or genocide.^2^^,^^6^^,^^19^, ^20^, ^21^, ^22^ A colonial process produces a dominant group that lives with privilege in a society, with systems designed to support their ways, and a colonized group who live in society, with systems designed to eliminate their ways and silence their voices.^20^^,^^22^, ^23^, ^24^ Settler-colonial consciousness emerges, which in the Canadian colonial state involves systems designed to privilege White, settler, and Western ways, and elevate White race superiority. Listening to Indigenous voices and knowledge, along with exploring ways to work with both Indigenous and biomedical perspectives to inform practice, policies, and research, is required to rectify epistemic racism; all are acts of reconciliation.

In this article, Indigenous people, their hearts, and cardiovascular health are discussed from diverse knowledge bases. We begin with introducing the coauthors, including who we are and our previous relationships. Then we set the stage with a discussion of Indigenous people in Canada, colonization, and related government strategies, noting the importance of key documents that are sources of knowledge that “un-silencing” Indigenous voices and acknowledge their thanks. Next, we critically reflect upon biomedical and social science approaches, to understand Indigenous people’s heart health and disease. Then we share insights from 2 Canadian Institutes of Health Research (CIHR)-funded studies concerning Indigenous people and heart health that were completed over the past 7 years. Both studies privileged Indigenous people’s voices, experiences, and knowledge. Drawing on all of these voices and perspectives, we discuss ways to inform practice, policy, and research within the field of cardiology. We offer guidance for steps toward reconciliation as a self-reflective, personal action, and as a collective professional action.

## Coauthors Locales

Over the past 7 years, the coauthors have collaborated on a mixed-methods research study funded by CIHR; the study was titled Debwewin—The Truth of Our Hearts. As well, the authors have individually worked in diverse capacities within healthcare systems as researchers, clinicians in community and acute care settings, decision makers, and community-based and postsecondary educators for decades. The lead author, Annette Schultz, is a White settler from Alberta, with French and German ancestry. She now resides on Treaty 1 lands and the homeland of the Métis Nation and is a professor in the College of Nursing, University of Manitoba. Thang Nguyen is a Vietnamese refugee who arrived in Canada in the late 1970s. He resides and works on Treaty 1 lands and the homeland of the Métis Nation, but he also serves on Treaty 2, 3, and 5 lands as a cardiologist for Cardiac Sciences Manitoba and the University of Manitoba. Moneca Sinclaire Nēhinan (Cree) has worked in Indigenous health for several decades and lives on Treaty 1 lands and the homeland of the Métis Nation. Her understanding of health is viewed through a person’s relationships to the land, as well as to self and others. More important to her view is that land–knowledge relationships must be handed down from generation to generation. Randy Fransoo is of Belgian ancestry and has lived most of his life in Winnipeg, which is Treaty 1 territory and homeland of the Métis Nation. He is Assistant Professor of Community Health Sciences and has conducted epidemiologic studies on cardiac patients in Manitoba. Elizabeth McGibbon is a White settler of working-class Irish heritage and a professor in the Rankin School of Nursing, at St. Francis Xavier University. She lives in Mi’kma’ki, the ancestral and unceded territory of the Mi’kmaq people. The research we have collaborated on concerning First Nations heart health aimed to diversify our understanding of health disparities by disrupting the dominance of biomedical perspectives stemming from Western worldviews.

## Indigenous Peoples: Layered Foundational Truths

In this article, we primarily use the term “Indigenous people,” rather than the constitutionally imposed term “Aboriginal people.” The exception is when citing another author’s work, for which we follow their language. Canadian-imposed colonial practices reach back over 400 years, and although specific strategies have evolved over time and vary across Canada, the result among Indigenous people has been social and material inequities that also have threatened their lives and health status for many generations.^20^^,^^25^^,^^26^ Colonial practices are systems of oppression that privilege being White and settlers’ voices and ways, while dismissing the voices, personhood, experiences, and knowledge of Indigenous people. Historical and current-day systemic racism is an embedded reality within Canadian healthcare systems.^16^^,^^19^, ^20^, ^21^, ^22^^,^^27^^,^^28^ Although these ideas are not news for Indigenous people, the same likely cannot be said of many White, settler, non-Indigenous people in Canada. Over recent decades, the truth of racism (see Box 1) within healthcare is revealing itself through public sharing of Indigenous people’s experience (ie, that of Brian Sinclair and Joyce Echaquan),^27^^,^^29^ and the Truth and Reconciliation Commission of Canada (TRC) report.^22^ Turning away in the face of racism is increasingly becoming an impossibility. Addressing racism and taking action toward reconciliation are important for any settler, but for healthcare professionals, taking personal and collective professional responsibility and action can and will save lives. Truth-telling and education regarding the colonial histories shaping our Canadian systems are initial steps to support reconciliation.

### Indigenous people in Canada: population counts

In Canada, Indigenous people encompass 3 distinct groups that constitute approximately 4.9% of the Canadian population, including 2.8% who identify as First Nation, 1.7% as Métis, and 0.2% as Inuit.^30^ Table 1 describes population counts of Indigenous people (First Nation, Métis, Inuit) for each province and territory, demonstrating that although the national percentages are small, there is variance in the numbers and population makeup across Canada.^31^, ^32^, ^33^, ^34^, ^35^, ^36^, ^37^, ^38^, ^39^, ^40^, ^41^, ^42^, ^43^, ^44^, ^45^, ^46^, ^47^ In addition, the median ages of all distinct groups of Indigenous people are younger than provincial median ages—in most areas by about 10 years.^31^, ^32^, ^33^, ^34^, ^35^, ^36^, ^37^, ^38^, ^39^, ^40^, ^41^, ^42^, ^43^, ^44^, ^45^, ^46^, ^47^ Population growth among Indigenous people is 4 times higher than that among other Canadians.^30^

**Table 1:** Counting Indigenous people across Canada based on 2016 Canadian Census

Territory or Province	Nunavut	NWT	Yukon	BC	AB	SK	MB	ON	QB	NB	NS	PEI	NL & Labrador
Provincial/territorial population of Indigenous people. n	30,550	20,860	8195	270,585	258,640	175,015	223,310	374,395	182,890	29,380	51,495	2740	45,725
Indigenous people, % of provincial/territorial population	85.9	50.7	23.3	5.9	6.5	16	18	2.8	2.3	4	5.7	1.9	8.9
Provincial/territorial Indigenous people, % of Indigenous people in Canada	1.8	1.2	0.5	16.2	15.5	10.5	13.3	22.4	10.9	1.8	3.1	1.6	2.7
First Nations people, n (%)of Indigenous people in province/territory	190 (0.6)	13,185 (63.2)	6690 (81.6)	172,520 (63.8)	136,585 (53)	114,570 (65.5)	130,585 (58.5)	236,680 (63.2)	92,655 (50.7)	17,575 (60)	25,830 (50.2)	1875 (68.4)	28,375 (62.1)
Métis, n (%) of Indigenous people in province/territory	165 (0.5)	3390 (16.3)	1015 (12.4)	89,450 (33.1)	114,375 (44.2)	57,880 (33.1)	89,360 (40)	120,585 (32.2)	69,360 (37.9)	10,200 (34.7)	23,310 (45.3)	710 (26)	7790 (17)
Inuit, n (%) of Indigenous people in province/territory	30,140 (98.7)	4080 (19.6)	225 (2.7)	1615 (0.6)	2500 (1)	360 (0.2)	610 (0.3)	3860 (1)	13,945 (7.6)	385 (1.3)	795 (1.5)	75 (2.7)	6450 (14)

AB, Alberta; BC, British Columbia; MB, Manitoba; NS, Nova Scotia; NB, New Brunswick; NL, Newfoundland; NWT, Northwest Territories; ON, Ontario; PEI, Prince Edward Island; QB, Quebec; SK, Saskatchewan.

Among First Nations people living on reservations and Inuit people living in northern communities, the majority report being able to converse in an Indigenous language, and of these people, the percentage who report an ability to speak their language as adults is higher than that who indicate their first language is an Indigenous one.^31^, ^32^, ^33^, ^34^, ^35^, ^36^, ^37^, ^38^, ^39^, ^40^, ^41^, ^42^, ^43^, ^44^, ^45^, ^46^, ^47^ In terms of cardiovascular disease, the significance of this growing rate of language acquisition is cited in research from Alberta and New Zealand, which notes that Indigenous communities with stronger connections to traditional languages also had lower rates of type 2 diabetes.^48^^,^^49^ Globally, lower rates of type 2 diabetes are reported among Indigenous populations in which traditional lifestyles were maintained, compared to the rates among those shaped by colonization and global economic and social forces.^50^ Although centuries of colonial strategies rooted in racism and genocide have inflicted harms and traumatized Indigenous people,^13^^,^^15^^,^^16^^,^^20^, ^21^, ^22^^,^^27^ increasing evidence shows a resurgence of Indigenous identity, voices, and knowledge.^13^^,^^14^^,^^16^^,^^20^, ^21^, ^22^^,^^24^^,^^25^^,^^51^ Although continuing to face various forms of racism, Indigenous people increasingly are standing up for their own truths and rights.

### Indian residential school truths

**Figure 1:**
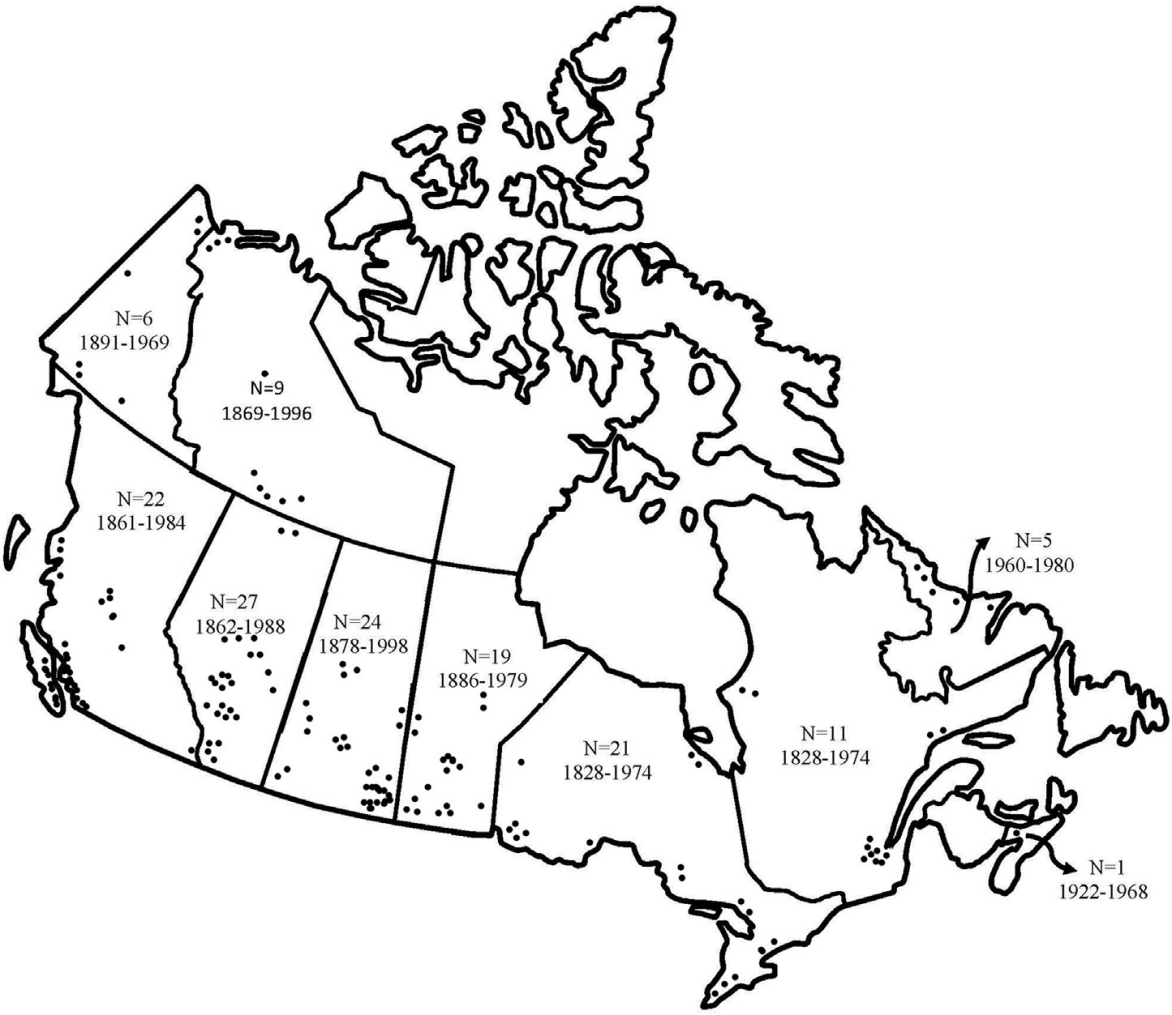
Mapping of Indian residential schools across Canada, which were in operation from the late 1820s until 1998. The network of 148 institutions spanned all Canadian provinces and territories.

Many readers have heard of residential schools and the fact that at least 150,000 children were forcibly removed from their homes, some as young as 2 years.^22^ Beyond the incredible heartbreak of this violating act, important to note is that these institutions were not educational schools. Rather, there were 148 Canadian institutions that served as a genocidal network funded by the government and administrated by religious organizations that were in operation from the 1820s through to closure of the last one in 1998 (Fig. 1). Equally important to note is that the targets of this network of institutions were Indigenous children (never settler or White children) who, after being forcibly removed from their homes, were detained in these institutions where cultural genocide was a stated intention, and where physical, sexual, mental, and spiritual abuses were inflicted upon them, as were acts of genocide. Recently, people living in Canada learned the truth of 215 unmarked graves of children who died at the Kamloops, British Columbia, Indian Residential School,^52^ followed by news of another 104 unmarked graves at an institution near Brandon, Manitoba, and 751 unmarked graves at an institution near Cowessess, Saskatchewan.

Those who have known of these graves for decades suggest that many more such announcements will be made as the grounds of other institutions undergo ground-penetrating radar scans.^53^ Two documents that shed light on these traumatic truths and others resulting from government-imposed structures of oppression are the Royal Commission on Aboriginal Peoples report^21^ and the TRC report.^22^ Notably, both are written by and from the perspectives and voices of Indigenous people about their experiences living within a colonial state intent on silencing them (see Box 2). For (at least some) Indigenous people, these documents created hope that their voices would be heard, after centuries of being pushed to the margins through being silenced and not being believed. For settler and White people, the facts can be hard to hear and may even seem unbelievable, yet, all these accounts reveal the truth of Canada’s colonial past and present.

Box 2 **Dr Sinclaire reflects on Indian residential schools (IRSs) and Indian hospitals (tuberculosis sanitoria)**

When the story of watching our children being forcibly removed is told, a variety of reactions have been heard, from, “I cannot imagine this happening to me, I would want to assassinate those people who took them away” to “This is horrible, but this happened so long ago.” Sadly, the last residential school closed in 1998 and the first opened in 1861; that means 3 generations of imposed genocide, and one generation without IRSs. As a side note, Indigenous children continued to be taken away through the 1960s through to the present day of social agencies removing children from “unfit homes.” Thinking of my own maternal grandparents who died from what biomedicine identified as coronary disease, age 52 and 54 years, or death from “broken hearts” due to seeing 5 of their children taken away to residential schools from ages 5 to 16 years. Having to witness these children returning back as “shell shocked” people, and then my grandparents continuing to see their grandchildren being raised in harsh ways that their children learned while in the IRS. Three of these children also went to a sanitorium for 6 to 10 years because they caught tuberculosis in the IRS. This story is not a unique story, but it is a story that is told from many children and grandchildren who are now beginning to understand the truth of how much “heart” pain their parents and/or grandparents experienced in an IRS.

### Truth of Indian hospital systems

This race-based system began in the 1890s, with hospitals run by missionaries, commonly situated in Indigenous communities.^54^ With the rise of tuberculous in the 1930s, the official Indian hospital system was formed, and the number of hospitals grew. In the 1960s, with the birth of Medicare, the Indian hospitals began to close, and the government closed most of them during the 1980s. Our search for the number and locations of Indian hospitals resulted in some findings but nothing we can report with certainty. Still, we know these hospitals existed across the country, and in 2018 a class action lawsuit was filed on behalf of Indigenous people who were patients at one of 29 Canadian Indian hospitals. Indigenous people who became patients in these hospitals received experimental treatments, some of a brutal nature, and often suboptimal care. There is also evidence of forced sterilization. For more information about Indian hospitals in Canada, additional reading is available.^54^^,^^55^

### Summarizing the layers of foundational truths

There are 3 central reasons for bringing the foundational truths and facts of the Indian residential schools and Indian hospital systems into our discussion. First, these truths provide evidence for understanding why healthcare encounters are experienced as unsafe, and the lack of trust among Indigenous people within healthcare settings.^13^, ^14^, ^15^, ^16^^,^^19^, ^20^, ^21^, ^22^^,^^27^^,^^56^ Learning about these 2 raced-based systems—which traumatized Indigenous people for centuries—is required to support health professionals, policymakers, and researchers in understanding Indigenous people’s sense of being unsafe within the healthcare system. Although these 2 raced-based structures are rooted within historical colonial actions, their reality and influence reach into current-day circumstances.^13^, ^14^, ^15^, ^16^^,^^19^, ^20^, ^21^, ^22^^,^^27^ Second, for settlers and White people, hearing these foundational truths sheds light on the privilege of their lives^23^^,^^57^; for them, healthcare systems largely function not as a threat, or as intentionally designed to silence their voices and discount their experiences, but rather to support their ways and voices. Third, stress levels inflicted upon Indigenous people through these systemic and epistemic racial actions have had and continue to have negative health effects,^13^, ^14^, ^15^, ^16^^,^^19^, ^20^, ^21^, ^22^^,^^27^ and as Dr Young noted, on heart health particularly.^1^ Health professionals’ awareness of these foundational truths will aid them in understanding the lack of safety within healthcare systems, and the traumas and racism that must be addressed to provide safe care. Acts of reconciliation to support improved health and healthcare delivery require seeing and knowing from perspectives and knowledge that reside outside White-race, settler-consciousness, and biomedical perspectives.

## Indigenous People, Cardiovascular Health, and Healthcare Accessibility: Reflecting on Western Perspectives From the Biomedical and Social Sciences

Our discussion of biomedical and social sciences is illustrative of evidence and perspectives concerning Indigenous people in Canada and cardiovascular health, informing critical reflections and questions about Western approaches. Health researchers report that the incidence of cardiovascular disease is at least 2 times higher among Indigenous people (11.5%), compared with that among non-Indigenous people (5.5%).^14^ The diseases commonly being referred to are diabetes, stroke, and myocardial infarction. Age-standardized mortality among First Nations men is about 30% higher, and among women 76.5% higher, in comparison to that in the general population.^2^ In our study titled Debwewin—The Truth of Our Hearts, the focus was on diagnostic angiograms as an entry point into cardiovascular care. We explored risk factors, related treatments, and access to care, along with health outcomes,^9^, ^10^, ^11^ and like many others have done, we compared First Nations (Indigenous people) with other people (predominantly non-Indigenous); this comparison revealed higher rates of illness, sex-based differences, and greater disease burden among First Nations people, in comparison to all other Manitobans.^9^, ^10^, ^11^ Epidemiologically defined disparities are useful evidence, but what to do with this awareness is our current quandary.^15^^,^^58^ That is, what questions need to be asked, and how do we understand and act on evidence that demonstrates the existence of health disparities and inequities?

### Epidemiologically defined disparities

We offer reflections on 2 critical questions concerning the approach of comparing Indigenous people with general (primarily White and settler) populations. In Canada, studies using health administrative data sources are commonly limited to reports on “status” First Nations people,^9^ due to lack of collection of race- or ethnicity-based data relating to healthcare,^59^, ^60^, ^61^ thereby limiting investigation of cardiovascular health among the 3 groups of Indigenous people (First Nations, Métis, and Inuit) living in Canada. Furthermore, people who identify as Indigenous, but who are not registered with Indigenous Services of Canada (meaning they do not have a status number), are counted as part of the general (non-Indigenous) population. Although the number of “non-status” Indigenous people may be small, this remains an example of erasing the records of Indigenous people within studies that attempt to report on their health status.

The second question concerns asking ourselves why we compare Indigenous people with populations of primarily White settlers.^62^ As we noted in the previous section, the historical and current-day realities of Indigenous people living in Canada is different than those of White settlers, in the context of a society structured by systems that privilege those who are White, and Western ways, and with continued oppressive marginalization of Indigenous people. As heard in Dr Sinclaire’s comment, we are studying the effects of colonization, rather than learning about Indigenous people themselves. In contrast, a recent study reported on an investigation involving 8 diverse First Nations communities,^2^ demonstrating a disruption in the tenacious tendency of locating White and settler health and outcomes at the center of focus, from which everything else is compared and evaluated. The study unsettled the elevation of being White and having settler status is what all ought to achieve. This ongoing comparison is an example of epistemic racism, with research methods that dismiss and invalidate Indigenous knowledge and voices in the production of evidence to inform healthcare system decisions.

### Health disparities and risk factors

Next, we reflect upon the reporting of cardiovascular risk factors, which commonly focus on individual-level factors. Once a greater burden of disease is identified, there is a discussion of cardiovascular risk factors that commonly include rates of obesity, hypertension, hyperlipidemia, atherosclerosis, diabetes, diet, exercise, tobacco use, and poverty. Reported rates are usually higher among Indigenous people in comparison to other (non-Indigenous) people, which then informs strategies to assist individuals in addressing the high rates of risk factors.^5^^,^^10^^,^^11^^,^^13^ McGibbon and colleagues^15^ suggest an alternate follow-up to the evidence of higher rates among a group of people—asking the question ”Why is it that this particular group has higher rates of risk factors in the first place?” That is, what are the root societal causes that result in higher risk factors among particular groups. This question refocuses our gaze to ask different questions to inform research. For example, Anand and colleagues’ study of 8 First Nations communities investigated protective factors, other than the common cardiovascular risk factors, such as trust among neighbors in the community, education, level of social support, and ability to obtain and take prescription medication.^2^

Indigenous methodologists have called for the inclusion of and privileging of Indigenous people—their voices and ways—in designing research, including identifying research questions, defining variables to be studied, and interpreting findings to support research methods and evidence that move beyond reliance on Western views and methods.^24^^,^^62^ Anand and colleagues offer a clear example of possibilities when this call is addressed by the research team. A second example in Canadian health research is the association between language and rates of type 2 diabetes,^48^ which has also been reported in other countries.^49^^,^^50^ The evidence suggests that in communities with higher rates of Indigenous language acquisition, there are lower rates of diabetes. This original research using language and cultural identity speculated that language is a proxy for identity, and owning Indigenous identity.^63^ Learning an Indigenous language(s) comes with teachings and relations with others, the land, and the creator—knowing the language is more than simply learning how to say words. This demonstration of resilience and resurgence appears to promote health, in comparison to living in shame, unsettled, and disconnected from Indigenous identity.

### Expanding how access to care is studied

Access to healthcare has also been investigated in response to identified health status differences. Once again, examination of the access to cardiovascular treatments, in particular when using health administrative data sets, commonly focuses on comparing First Nations people with other populations. The Debwewin study explored rates of diagnostic angiograms, hospital admissions, and follow-up appointments with physicians.^10^ As reported in other studies, evidence suggests that First Nations people have lower rates of healthcare engagement, compared with those of all other Manitobans, with the exception of higher rates of hospital admissions and readmissions.^10^ In the community study conducted by Anand and colleagues,^2^ they reported the following examples of lower levels of access to care: reduced primary care access, higher incidence of emergency room visits, and difficulties in filling prescriptions (despite “drugs being covered”). Beyond noting the trend and difficulties of comparing Indigenous people with others (predominately White people), another area to critically reflect upon is how we understand and study access. Horrill and colleagues^64^ outline an understanding of access from a biomedical perspective that commonly focuses on geographic location, availability of services and providers, retention of providers, and financial considerations. They also outline an understanding of access through a postcolonial perspective, which focuses on previous negative experiences in healthcare (racism, discrimination, fear of judgement or not being believed), language barriers, and ongoing colonial relations within healthcare.^64^ Given the historical and ongoing colonial structures and racism within healthcare in Canada, exploring access through a postcolonial perspective reveals useful evidence to guide practice, policymaking, and research to address unsafe contexts within healthcare, thus improving access to care and quality of care delivered.

### Summarizing challenges with Western perspectives

“… cardiovascular disease is an all too logical result of the stark realities of long-term racism-related stress and intergenerational traumas. Colonialism continues; it does not somehow go away because we think these atrocities happened in the past. Generational family trauma transmissions continue because adults’ traumatic experiences and their impacts become transmitted within family and community systems across generations. There is remarkably little literature about the ways in which oppression-based trauma becomes threaded through generations of oppressed peoples.^15^ (p.140)

We challenge health researchers, healthcare decision makers and policymakers, and health professionals to consider how we personally and collectively can learn more about colonization and the stress of oppression, and explore pathways for personal, professional, and systemic change. This change involves acknowledging and reflecting upon privilege and how racism and discrimination are not common day-to-day experiences in the lives of many healthcare professionals. As awareness of privilege grows, so does capacity to challenge societal structures and systems that result in racism and acts of discrimination.^23^^,^^57^ Although awareness is growing through stories of the Indian residential schools and Indian hospitals, our learning must also include hearing the impacts of these colonial and genocidal institutions. For example, reread and listen to the story shared in Box 2, and then reflect on how your understanding of Indigenous people has been (re)informed. As the TRC calls to action^65^ note (see Box 3), those in healthcare leadership roles and those who can make a difference need to change their actions.

Box 3 **Truth and reconciliation calls to action—health-related actions**

Abbreviated Calls to Action 18 to 24^65^ (pp. 2 and 3 for original)

18. Acknowledge the current state of Aboriginal health in Canada is a direct result of previous Canadian government policies, including IRS (Indian residential schools), and to recognize/implement the health-care rights of Aboriginal people as identified in international law, constitutional law, and under the Treaties.
19. Call upon the federal government to collaborate with Aboriginal peoples, to establish measurable goals to identify and close gaps in health outcomes among Aboriginal and non-Aboriginal peoples. Such indicators may include, infant mortality, maternal health, birth rates, infant and child health status, ***chronic diseases***, illnesses and injury incidence, and ***availability of appropriate health services.***
20. In order to address the jurisdictional disputes concerning Aboriginal people who do not reside on reserves, we call upon the federal government to recognize, respect, and address distinct health needs of Métis, Inuit, and off reserve Aboriginal people.
21. Funding for existing and new Aboriginal healing centers to address the physical, mental, emotional and spiritual harms caused within IRS.
22. We call upon those who can effect change within the Canadian healthcare system to recognize the value of Aboriginal healing practices and use them in treatment of Aboriginal patients in collaboration with Aboriginal healers and Elders where requested by Aboriginal people.
23. Call upon all levels of governments to:
  • Increase the number of Aboriginal professionals working in the healthcare field
  • Ensure retention of Aboriginal healthcare providers in Aboriginal communities
  • Provide cultural competency training for all healthcare professionals
24. Call upon all medical and nursing schools in Canada to require all students take a course dealing with Aboriginal health issues, including the history and legacy of IRS, the United Nations Declaration on the Rights of Indigenous Peoples, Treaties and Aboriginal rights, and Indigenous teaching and practices. This will require skills-based training in intercultural competency, conflict resolution, human rights, and anti-racism.

## Listening to Elders and Knowledge Keepers’ Stories of the Heart and Caring for Our Hearts: Indigenous Voices and Perspectives

**Figure 2:**
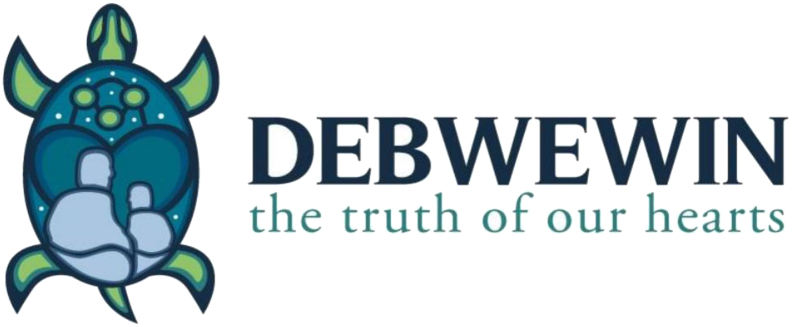
Debwewin study logo was designed by an Indigenous Multimedia Creative Dwayne Bird. The turtle represents, as in the Anishinaabe teachings, the sacred teaching of truth. The back of the turtle uses silhouettes of people overlaid on a heart. This is a representation of the people involved in listening/looking and discovering the truth of why Indigenous peoples suffer from heart disease.

We now turn our attention to spaces that privilege Indigenous voices, experiences, and science (ways of knowing and doing). Since 2014, Dr Schultz has led and co-led 2 CIHR-funded studies that involved Indigenous people interested in both heart health and caring for their hearts. As previously stated, the Debwewin study^10^ was a 5-year mixed-methods study involving the coauthors of the current article (Fig. 2). The second was a 2-year digital storytelling study led by Dr Lorena Sekwan Fontaine, entitled *mite* *achimowin (Heart Talk): First Nations Women’s Expressions of Heart Health*.^14^ Dr Sinclaire was also involved in this study. In this section, a brief summary is provided for each study, along with links to a video and podcasts from the Debwewin study, and a link to the digital stories and other study materials from the mite achimowin study. Finally, Dr Sinclaire’s reflections on the 2 studies (Box 4) supplement the various study descriptions.

Box 4 **Dr Sinclaire reflects on Mitah/Mite teachings**

Mitah (heart: Cree)

I have heard numerous Elders or Knowledge Keepers speak about the heart (Mitah—Cree) as one of the sacred teachings. I was reminded of this when I heard David Blacksmith, Knowledge Keeper, in the film Pac-Ow-Tay: Our Beating Hearts spoke about how the body cannot function without the heart. I remember when Blacksmith said this I started to think about how when people are pronounced brain dead, they are kept alive through a respirator that allows their heart to continue to beat; but once they are taken off the respirator, they pass away.

Esther Sanderson, an Elder, in the research project mite achimowin, in her video Mite Mekewin, explained how the heart is recognized as having two functions—it is an organ in our body that pumps blood throughout the body and secondly she says, “the heart is where the blood that flows through her body carries her ancestral knowledge, ceremonies, songs … it is the same blood flowing that makes me who I am today.” It is the second description of heart that contains the teaching of how the heart is more than an organ—it is a vehicle that carries not only the blood but also blood memories or DNA from all our ancestors. And it is these blood memories from the heart that have enabled us as Indigenous peoples to continue despite colonialism. This was also a teaching I received in the mid 1970s at the Indian Ecumenical Conferences in Morley, Alberta. When I was at Morley, these words resonated with me; however, through time and ongoing listening, this teaching makes greater sense of how important the heart is to carry the message of “never giving up” who we are as a people. I think about all my relations that never gave up on who they were and it is this perseverance that has continued to beat through my heart and is wanting me to continue the same story for the next generation.

Mary Wilson, one of the Wisdom Keepers in the film Pac-Ow-Tay: Our Beating Hearts spoke about how the heart is the seed of humanity, it is the center of life; it’s the first thing. She explains how the heart is also where our voice comes from—it doesn’t come from our head; it comes from our heart, and when our heart speaks, it is our spirit. Blacksmith also talked about how mitah can be seen in the word mita’yun, meaning tongue in Cree, indicating how important it is that when one speaks, it must be from the heart. I have also heard that the longest journey one takes is from the head to the heart; for me, this means when we speak from what we have experienced, and not just from books, we begin to speak from the heart. And if we speak from the heart, it allows us to be able to transmit knowledge in a good way to other people who may need guidance and understanding. Mary says, “the heart kind of talks to us to where we need to be as a people.”

Mary continues to speak about how if the center of life or life force gets disrupted, then the heart becomes weaker. For Indigenous peoples, these disruptions include historical trauma from residential schools, racism, loss of whole populations from disease, forced starvation, and so on. Blacksmith also spoke about how the heart is a living being and how when the heart gets broken, it affects your health. In the digital video series, Eliza Beardy in My Heart Beat, explained how her heartbeat was passed down from her mother and how her mother got her heartbeat from her mother, and so on, but also that her relationship to her family is her heartbeat. Beardy talked about how her parents watched their children being taken away to attend residential schools. Beardy wondered, “How many times her parents’ hearts were broken when the children were taken away to go to residential school?” The questions about how many times our hearts have been broken were the same questions that I had heard when I was in Morley, Alberta. These Knowledge Keepers explained that although we have gone through much with the arrival of Europeans, it was our kind hearts that would keep us strong as a nation.

Kind hearts, they explained, were about treating our people who are suffering with kindness, and about always remembering to pray for those that have lost their path in Pimatisiwin (living the good life: Cree). The elders/knowledge keepers spoke about how our hearts would be strong again once we get back to ceremony, dancing, singing, and speaking in our language. Heart for Indigenous peoples becomes healed from not only exercise and eating right but also it is about having ceremony and loving people around you.

Kachina Blacksmith, daughter of Wisdom Keepers Sherryl and David Blacksmith, spoke about how the young people had sweats, ceremony, for both her grandmother and her father, when each was suffering from broken hearts from someone significant passing away. She mentioned that, while the medical profession was looking after them physically, she and her sibling where looking after their spirit, to ensure their hearts remained kind. Edward Eastman, Wisdom Keeper, also spoke about how important it is to talk to one another when you are feeling lonely and depressed.

This view that the heart is a living being is reiterated in the podcast Transformation of Knowledge when Hildebrand, a first-year medical student, was very surprised that how the heart was understood in medical school was not the same as what she was hearing from the Wisdom Keepers. Hildebrand spoke about how she learned from the Wisdom Keepers that speaking in a kind manner to people is using your heart in a good way, and how she feels it is important to remember this, especially in working with Indigenous people. In the same podcast, Kaltenblaugh, a nursing student, and research assistant for the Debwewin study speaks. He went through medical records, where he reflected on how non-Indigenous healthcare professionals may unknowingly “mistreat Indigenous peoples through their own stereotypes in how they wrote about non-compliance in the patient’s medical chart, versus how for a non-Indigenous person may write, ’person having a bad day.’” He wondered if this affected Indigenous peoples’ hearts when they observed how they were being treated in comparison to non-Indigenous peoples (http://www.sbrc.ca/schultz/).

### Debwewin—The Truth of Our Hearts

Our discussion of the Debwewin study now focuses on the qualitative method, which originally was to interview First Nations cardiac patients to hear their stories regarding their heart, caring for their heart, and healthcare encounters. Although many team members are seasoned health researchers, we encountered difficulty with recruitment for interviews, which we had never had before. At a team meeting, the study Elder Mary Wilson spoke about how our approach was not following Indigenous ways. She asked 3 questions: (i) How are we creating relationships?; (ii) How are we caring for and offering back to the participants?; and (iii) How are we envisioning touching the lives of generations to come? Through that meeting, we shifted our method to creation of a 35-minute documentary film. Following Elder Wilson’s lead, we reached out to 4 Indigenous knowledge keepers to have an initial conversation with them about the Debwewin study. After a few meetings, we agreed to meet 4 times over a 1-year period (2017-2018). Each meeting was video-recorded by a local Indigenous production company (Code Breakers, Inc.), where Elder Wilson and the 4 knowledge keepers gathered with a few team members to have a conversation about the heart, caring for the heart, and Indigenous people’s interface with our healthcare systems. Each gathering ended with a feast.

Materials from recorded gatherings were co-produced into the documentary called *Pac-Ow-Tay: Our Beating Heart Stories.*^66^ Documentary materials were also produced into a podcast series, with 3 podcasts from documentary materials, and 2 based on interviews. Dr Sinclaire had conversations with 2 Indigenous health professional university students involved in the study (mentioned in Box 4 reflections), and an interview with Elder Wilson and Dr Schultz. Podcasts can be accessed here: http://www.sbrc.ca/schultz/.

The Pac-Ow-Tay documentary provides the opportunity to listen to Indigenous perspectives regarding the heart, which go beyond understanding the heart as just a physical organ. Stories that can be heard in the documentary include the following: teachings about the heart and traditional Indigenous practices; and 3 generations weaving traditional practices with biomedical treatment of heart disease with their family, along with discussing other historical and current-day experiences within North American society that shape their beating hearts. David Blacksmith, one of the knowledge keepers, spoke about how the heart can be broken when a spouse dies. and if the remaining spouse has no relationship with another living entity, such as grandchildren, a dog, or a horse, then that person will also soon pass away.

### mite achimowin (Heart Talk): First Nations Women’s Expressions of Heart Health

This name came through conversations among the team members involved in this study. The idea underlying the name was to document what Elders, grandmothers, mothers, and aunties would talk about regarding the heart and caring for the heart. Through a week-long workshop, team members worked with 6 women and an Indigenous production company, to support the women in developing their digital story.^14^ On the final day, we held a feast that included guests from their communities to share their digital stories. Four digital stories are publicly available^67^ on the National Collaborating Centre for Indigenous Health webpages, and can be accessed here. A report entitled *Understanding First Nations Women’s Heart Health*^13^ was also developed. The report explores the role colonization has played in current-day reported heart disease, risk factors, and treatment of First Nations women’s heart health, and considers strategies to address the disparities defined through health research concerning First Nations women’s heart health.

### Summary

Materials from the Debwewin and mite achimowin study provide readers access to spaces where Indigenous experiences and ways of knowing/doing are privileged, thereby elevating Indigenous voices and ways of speaking of the heart, caring for the heart, and interfacing with our healthcare systems, as various individuals share their knowledge about the heart and caring for the heart. These materials shed light on the reasons that the poor health outcomes experienced by Indigenous peoples in Canada must be understood in the context that health from an Indigenous perspective involves more than physical realms. Inequities in health among Indigenous peoples are prolifically documented from a biomedical perspective; however, the solutions to improve health must be understood within the legacy of colonial history and the social and political contextual barriers that continue to impact Indigenous people. Many Indigenous people do not want to eradicate biomedical approaches, but rather wish to work alongside the medical community to create Indigenous pathways to health that include Indigenous people’s voices and stories. Listening to Indigenous voices is an opportunity to hear these perspectives and reflect upon threads that connect to centuries of government-imposed colonial and genocidal strategies. Finally, these study materials demonstrate resilience and resurgence of Indigenous knowledge and methods.

## Cardiovascular Healthcare, Indigenous People, and Reconciliation: Building Relationships and Respect

Respect for different ways of understanding and living is at the core of any reconciliation activity. The complexity of entrenching this respect into action in all that we do should not be underestimated. We must challenge and transform the foundational disrespect for Indigenous ways of knowing and doing that was used to justify colonial policies and is persistently embedded in our laws, schools, health systems and unconscious minds.^68^*(p.261)*

**Figure 3:**
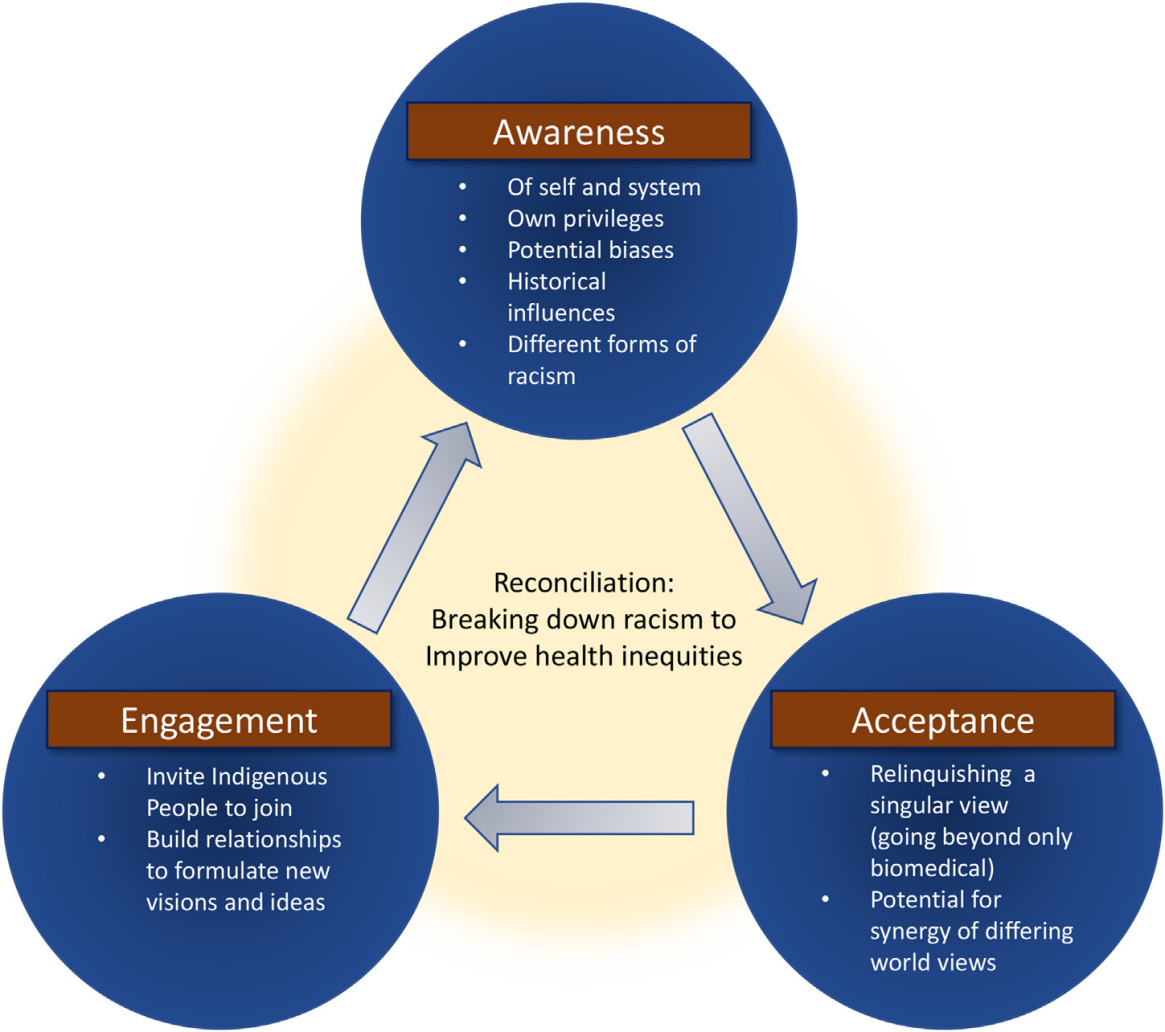
Reconciliation—realizing synergies arising from reflecting on one’s worldview and consideration of other worldviews, stepping into antiracist healthcare practices, and working with 2-eyed seeing approaches.

Our focus now turns toward healthcare practices, decisions, and research. Given the realities of the colonial and genocidal histories that have shaped our healthcare systems, and the demonstrated epistemic racism that influences production of evidence informing current-day healthcare decisions, simply refining a Western approach without redressing colonial histories is unlikely to address cardiovascular health disparities identified among Indigenous people. Rather, as Sinclaire and colleagues^18^ recently argued, there is a need to consider ways to move beyond working from a singular (biomedical) worldview by embracing multiple ways of understanding and approaching health. To be clear, the intent is not to dismiss Western or biomedical ways, but rather to share spaces and power in understanding health status in deriving strategies to improve health. As noted by Smylie^68^ above, this challenge needs to unfold on a personal level, collectively within professional contexts, and also within our healthcare and other systems. Three topics are discussed below to assist readers with engaging in the challenge through personal reflections on their own practices and perspectives, and taking steps toward reconciling epistemic and systemic racism within our professions and healthcare systems (Fig. 3). Each can be a starting point for change to disrupt our colonial ways within the healthcare arena, but none should be an endpoint for taking action.

### Our worldview and the weakness of tunnel vision

Dr Jon Tilburt, a Mayo Clinic physician, wrote about the professional weakness that comes from remaining unaware of one’s worldview, at least as it pertains to healthcare and strategies to address health inequities.^69^ He defines a worldview as a philosophy, which is a construct consisting of a set of beliefs and assumptions that inform how one makes sense of life that is commonly informed by experience.^69^ Within the arena of healthcare, the primary worldview at play is a biomedical perspective, which aligns with the design of our current healthcare system. Although many assumptions underlie a biomedical worldview, important foundations include the following: (i) beliefs in individualism (a focus on individuals, their behaviours, and pathophysiologies in the absence of social, political, cultural, and economic forces); and (ii) positivist beliefs in the possibility and desirability of objectivity and neutrality (beliefs about the apolitical nature of knowledge, including the possibility of eliminating bias).^18^^,^^70^

Tilburt and others argue that these beliefs and assumptions are underlying mechanisms that allow implicit bias to operate within practice and healthcare decisions, and that when left unquestioned, support practitioners, researchers, and policymakers in operating with limited awareness.^57^^,^^69^ As discussed earlier, an example is the biomedical approach of comparing Indigenous populations with general populations that consist primarily of White, settler populations. Although methodological guides are established regarding data collection and analysis that support claims of objectivity and reduced bias, there has been no accounting for an inherent bias within our healthcare systems (historical and current).^71^ As Dr Sinclaire asserts, studies of Indigenous people today will tell us more about the impact of colonization than about Indigenous people—that is, the impact of systems designed to silence and discriminate against Indigenous people. Tilburt goes on to say that the lack of awareness and humility on the part of individual practitioners and as a collective profession, regarding their worldview weaknesses, inhibit the ability to effectively address health inequities.^69^ This ability includes one’s ability to consider a different set of beliefs, assumptions, and worldviews, along with an ability to consider that perhaps one is not seeing the full picture. These considerations are essential to working effectively, and in particular, with those who are considered marginalized and racialized, whose worldview and experience often are not aligned with societal systems, such as healthcare. Building relationships with and respect for Indigenous people, their knowledge, and their worldviews is required to disrupt epistemic racism and other forms of racism within healthcare that underlie cardiovascular and other health disparities.

### Antiracist healthcare practice

Antiracist healthcare practice can take many important directions. Some points of entry include opening our eyes to seeing and acknowledging the pathways from past and current colonial oppressions, such as those described in the current article. The first step, which can be a tricky process, is becoming aware of and able to name our own privileges and hence our power within society and in healthcare encounters, which exist across race, gender, and social class, to name a few factors. With these insights, the next step is to understand how our privilege operates in everyday practice, even when we cannot see it.^57^ Connecting the pathways of oppression and privilege will help to identify policy drivers that are operating in discrimination occurring in the everyday lives of “others,” as well as the other side of the coin—in this case, White settler privilege (eg, institutional policies, professional practice policies, and public governance policies at the provincial/territorial and federal levels).

Being educated about and acknowledging these societal structures is an imperative foundation for antiracist healthcare practice. Finally, antiracist healthcare practice cannot happen without specific and measurable action: educating ourselves and our colleagues and communities, supporting others who speak up, and tackling racism at the policy level. Although education is important, it cannot, by itself, tackle racism because racism is systemic—created and sustained by social, cultural, political, and economic structures of society.^57^ As has been noted previously, these structures within a colonial state such as Canada support specific groups of people (White settlers) and aim to silence others (Indigenous people).

### Two-eyed seeing: teachings and possibilities

The final topic for discussion concerns the work of relinquishing the mindset of relying solely on evidence rooted in a biomedical worldview.^18^ In the fall of 2014, Mi’Kmaq Elders Albert and Murdena Marshall shared the Etuaptmumk teaching, which translates as “2-eyed seeing.”^18^^,^^72^^,^^73^ This teaching is not a research method, so it comes with no prescription of a process nor a “how to.” Rather, it is a way or process regarding how one conducts one’s life and business. At the core, this teaching embraces different ways of knowing and learning to see, by using strengths from an Indigenous eye (worldview), alongside the strengths from a biomedical eye (worldview). How each eye can contribute to understanding will shift from situation to situation; thus, this process does not lead to a static endpoint. Instead, adopting this process aids one to continually ask what is being heard and seen from both eyes. When anticipated moments of imbalance (or silencing of one perspective) arise, having a plan to support the seeking of insight from the silent eye is important.

**Figure 4:**
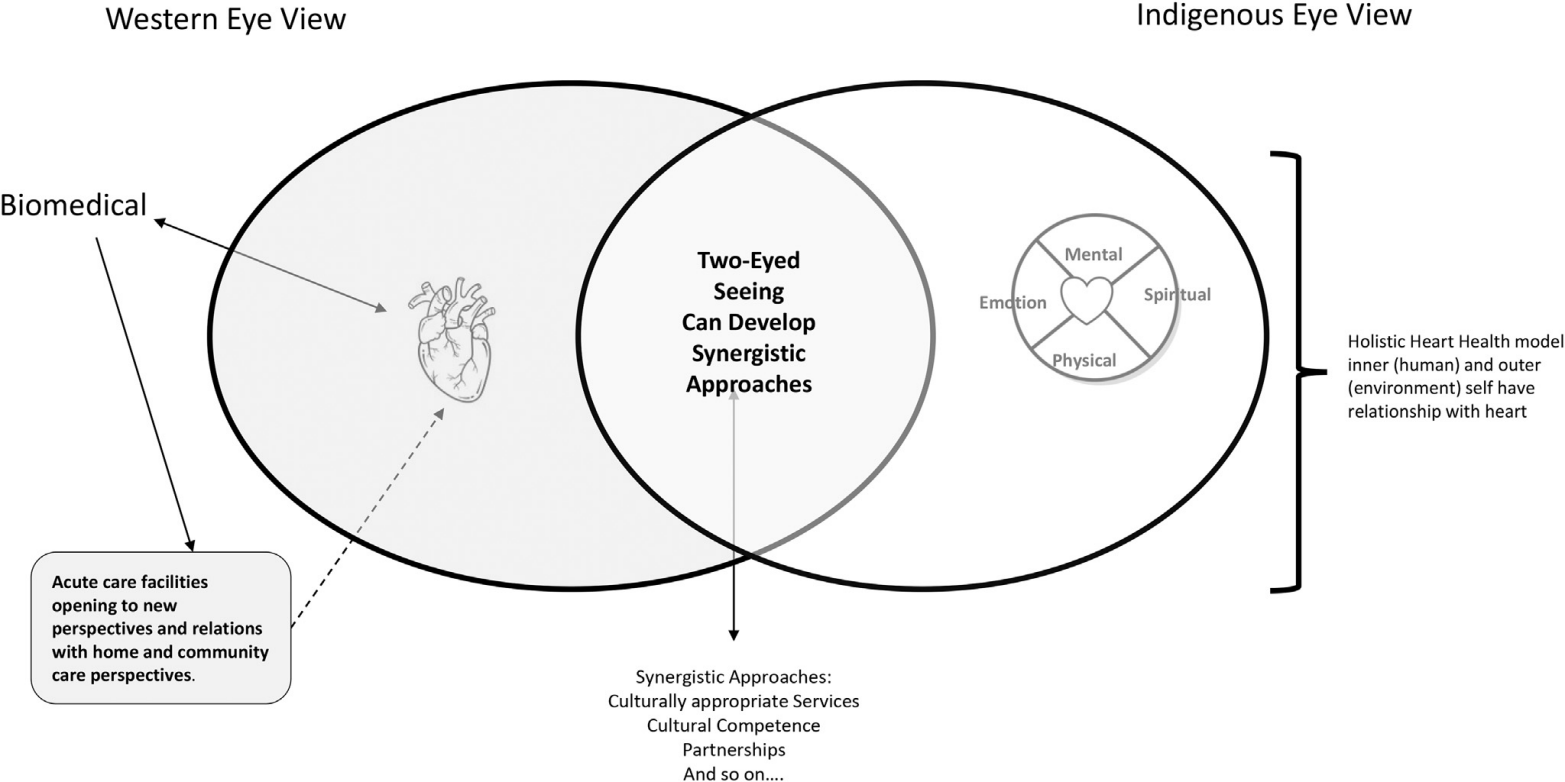
Two-eyed seeing, and opening to new ways of seeing and understanding.

By enhancing our collective ability to work together and across worldviews, our knowledge will broaden to strengthen our strategies to address areas of concern. For example, from an Indigenous eye viewpoint, knowledge will focus on relationships with others, but also with self, the land, and Indigenous identity; therefore, ways of proceeding might include reflection on current relationships, areas that require healing or the building of new relationships, and ceremony. Although 2-eyed seeing does not ask White settler practitioners, decision makers, or health researchers to become adept with knowledge from the Indigenous eye, the 2-eyed approach would encourage these healthcare leaders to consider ways to invite Indigenous people who have this knowledge to join in healthcare delivery and research. From a biomedical eye, some knowledge will focus on the individual’s body, risk (or other precipitating) factors, treatments (pharmaceutical, surgical, and other supportive actions for an individual), and barriers or facilitators concerning access to healthcare. From seeing and considering knowledge examples from each eye, one can begin to imagine possibilities for a new vision of how to address health inequities reported among Indigenous people (Fig. 4).

### Summary of building relationships

Our ability to see in new ways, and be open to learning, in this case about Indigenous people, their hearts, their heart health, and how to care for their hearts, will guide our actions forward. Such a process requires consideration of perspectives that move beyond how we currently sees and understand the world. Our current view, through the dominant lens of the Western biomedical worldview, in healthcare systems is not capturing the whole picture required to address cardiovascular health disparities among Indigenous people. Rather, reconciliation of the colonial nature of our relations, which in our current healthcare systems results in silencing of Indigenous voices, and repression of Indigenous experiences and knowledge, is imperative.

## Conclusions

At this point, take a moment to reflect upon the role historical and ongoing oppression (racism) has and is having on cardiovascular health among Indigenous people in Canada. As well, consider possibilities for individual and collective actions to redress the various wrongs. As an example, how can we disrupt the fact that evidence informing healthcare decisions continues to rely almost exclusively within Western biomedical perspectives, which means healthcare strategies are rooted in epistemic racial bias.^16^ Cardiovascular health among Indigenous people depends on acts of truth-telling and listening, as initial steps towards reconciliation, that are followed by actions of change to reconcile oppressive systemic racism impacting the health of Indigenous people.

This article provides much to reflect upon—not only truth-telling of required knowledge, but also how healthcare professionals’ worldviews and systems are designed with specific biases that privilege White settler and Western perspectives, subsequently discriminating against and silencing non-Western perspectives. Four offerings can guide actions of disruption regarding ongoing racism within healthcare.

1. Become familiar with the TRC Calls to Action, and act on them.
2. Become more familiar with antiracist healthcare practice, your own worldview, and how what you are seeing might not be a broad enough picture to effectively address health inequities in professional practice as well as professional organizations.
3. Become educated regarding how privilege is operating in your life, and actively start disrupting the ways that discrimination creates barriers in the lives of the people you serve, as well as in the lives of some of your colleagues.
4. Create relationships with, and find ways to start working with, Indigenous people.

All of these steps are acts of reconciliation, and moving in these directions is imperative to redress historical and continued colonial power and racism, which underlie the cardiovascular health disparities among Indigenous people, compared with White people, in Canada.^1^^,^^6^^,^^15^ Perhaps our response can be realization that it is time to listen to Indigenous people and actively work toward system change so that they move out of societal and system margins. For their cardiovascular health; yes, but also, we suggest, for the health of our society and shared lands.
